# Predicting the Accumulation of Ionizable Pharmaceuticals and Personal Care Products in Aquatic and Terrestrial Organisms

**DOI:** 10.1002/etc.5451

**Published:** 2022-09-23

**Authors:** Laura J. Carter, James M. Armitage, Bryan W. Brooks, John W. Nichols, Stefan Trapp

**Affiliations:** a School of Geography, Faculty of Environment, University of Leeds, Leeds, United Kingdom and Northern Ireland; b AES Armitage Environmental Sciences, Ottawa, ON, Canada; c Department of Environmental Science, Center for Reservoir and Aquatic Systems Research, Institute of Biomedical Studies, Baylor University, Waco, Texas, USA; d South Bohemian Research Center of Aquaculture and Biodiversity of Hydrocenoses, Faculty of Fisheries and Protection of Waters, University of South Bohemia in České Budějovice, Vodňany, Czech Republic; e Center for Computational Toxicology and Exposure, Great Lakes Toxicology and Ecology Division, Office of Research and Development, US Environmental Protection Agency, Duluth, Minnesota, USA; f Department of Environmental and Resource Engineering, Technical University of Denmark, Kongens Lyngby, Denmark

**Keywords:** Bioconcentration, environmental modeling, pharmaceuticals

## Abstract

The extent to which chemicals bioaccumulate in aquatic and terrestrial organisms represents a fundamental consideration for chemicals management efforts intended to protect public health and the environment from pollution and waste. Many chemicals, including most pharmaceuticals and personal care products (PPCPs), are ionizable across environmentally relevant pH gradients, which can affect their fate in aquatic and terrestrial systems. Existing mathematical models describe the accumulation of neutral organic chemicals and weak acids and bases in both fish and plants. Further model development is hampered, however, by a lack of mechanistic insights for PPCPs that are predominantly or permanently ionized. Targeted experiments across environmentally realistic conditions are needed to address the following questions: (1) What are the partitioning and sorption behaviors of strongly ionizing chemicals among species? (2) How does membrane permeability of ions influence bioaccumulation of PPCPs? (3) To what extent are salts and associated complexes with PPCPs influencing bioaccumulation? (4) How do biotransformation and other elimination processes vary within and among species? (5) Are bioaccumulation modeling efforts currently focused on chemicals and species with key data gaps and risk profiles? Answering these questions promises to address key sources of uncertainty for bioaccumulation modeling of ionizable PPCPs and related contaminants.

## INTRODUCTION

The extent to which chemicals bioaccumulate in aquatic and terrestrial organisms represents a fundamental consideration for chemicals management efforts intended to protect public health and the environment from pollution and waste. Basic and translational research, including professional practice activities, have contributed to the development of bioaccumulation science for inorganic and organic chemicals. Because empirical information for uptake and elimination is unavailable for the vast majority of the approximately 350 000 chemicals and chemical mixtures registered for global production and use (Wang et al., 2020), it is imperative to understand the processes that result in bioaccumulation and develop predictive computational models that account for these processes. Bioaccumulation models can be used to assess diverse exposure scenarios to quantify environmental risk and prioritize chemicals for control and interventions, including substitution by and design of less hazardous substances.

Most ingredients within pharmaceuticals and personal care products (PPCPs) are introduced to the environment from domestic and industrial wastewater treatment plant discharges and in poorly treated or raw sewage, with additional inputs from veterinary use (aquaculture and livestock production) and the land application of biosolids, effluents, or manure. The PPCPs encompass a diverse group of bioactive ingredients including human and veterinary medicines as well as chemicals in personal care products such as sunscreens, detergents, and disinfectants. For the present study, we also included per- and polyfluoroalkyl substances (PFAS), noting that although PFAS are not generally present in PPCPs except as impurities (Brinch et al., 2018; Fujii et al., 2013; Whitehead et al., 2021), their properties (e.g., strong acids, affinity for phospholipids and serum albumin) and known kinetic behaviors in fish can offer significant insights into the bioaccumulation of ionizable PPCPs.

Numerous chemicals in commerce, including the majority of PPCPs, are ionizable across environmentally relevant pH gradients (Franco et al., 2010; Manallack, 2007; Newton & Kluza, 2016). Despite recognition that pH influences bioavailability and toxicity of ionizable contaminants, empirical information for accumulation of ionizable PPCPs in ecological receptors was quite rare a decade ago. Initial observations of bioaccumulation of basic pharmaceuticals by fish in the field (Brooks et al., 2005; Ramirez et al., 2007), along with the development of predictive models for gill uptake of ionizable chemicals (Erickson et al., 2006a, 2006b) stimulated research activities in this area (Daughton & Brooks, 2011). In an earlier review of this topic, Fu et al. (2009) noted the important contribution of ionizable contaminants more broadly to chemicals in commerce. The paucity of attention previously given to ionizable chemicals, coupled with the lack of predictive understanding of the bioaccumulation potential of ionizable PPCPs in aquatic and terrestrial systems led to the identification of this question as a priority research need by Boxall et al. (2012): “How can the uptake of ionizable PPCPs into aquatic and terrestrial organisms and through food chains be predicted?”

In the present study we considered the challenge of predicting the bioaccumulation of ionizable PPCPs, recognizing that this outcome is a function of both uptake and elimination processes. For both aquatic and terrestrial systems, we examined key processes involved in the accumulation of ionizable chemicals by individual species and reflected on research developments over the past decade within a predictive modeling context. We concluded by providing recommendations for future research to improve bioaccumulation predictions of ionizable PPCPs, including identification of priority research questions for the next decade.

**FIGURE 1:**
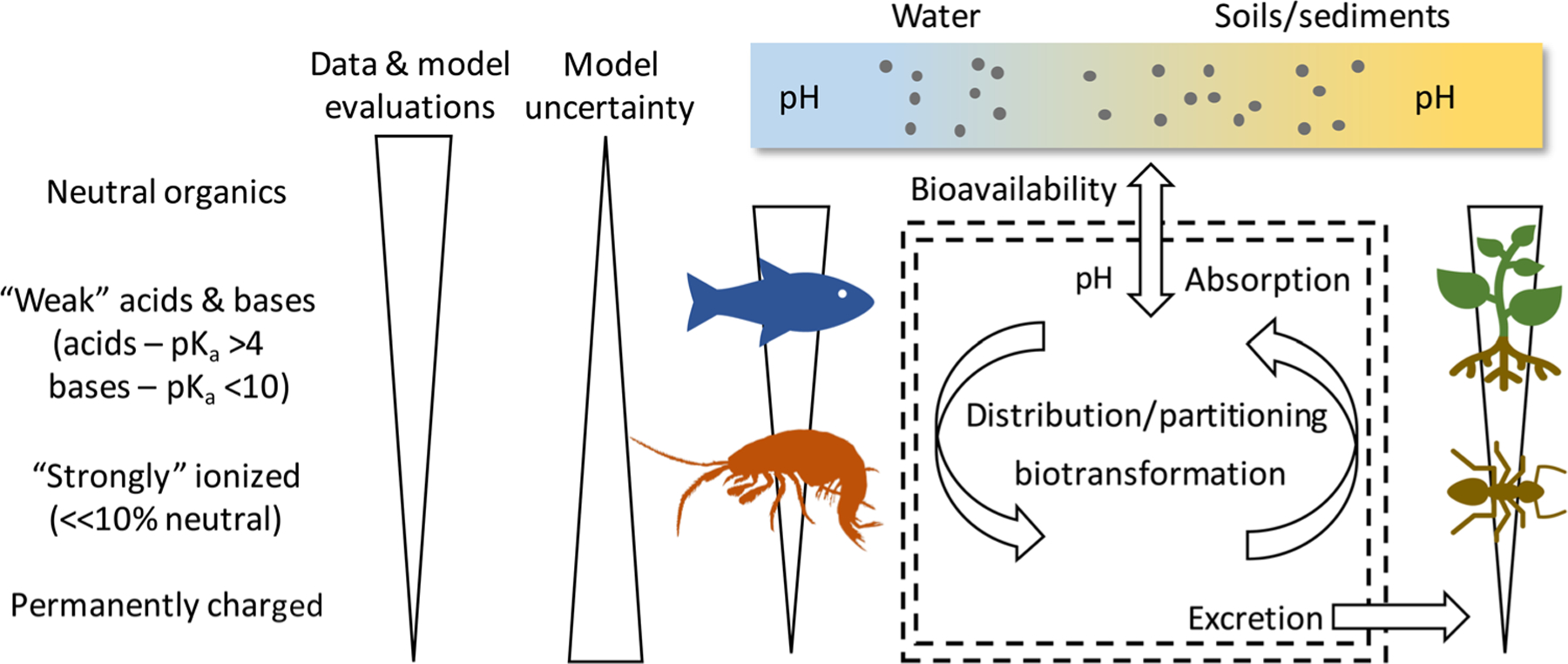
Chemical accumulation in living organisms as net result of absorption, distribution, metabolism (biotransformation), and excretion processes. Knowledge status for different chemical groups.

Chemical accumulation in living organisms occurs as a net result of absorption, distribution, metabolism (biotransformation), and excretion processes (ADME; Figure 1). Excluding higher vertebrates, current understanding of these processes in ecological receptors is greatest for fish and plants; however, conceptually similar processes can be expected to apply to other taxa including aquatic and terrestrial invertebrates. Common to all ADME processes is a need for chemicals to transit one or more biological membranes. Neutral chemicals bound in the environment (e.g., to dissolved or soil organic material) or within an organism (e.g., to plasma proteins or root tissue protein) cannot diffuse across membranes. For such chemicals, the direction and magnitude of the diffusion gradient is determined by “unbound” (and therefore bioavailable) chemical concentrations on either side of the membrane. For weak acids and bases, the membrane permeability of the neutral form greatly exceeds that of the ionized form. If one assumes that the ionized species does not diffuse across the membrane, the bioavailable fraction is determined by the extent of ionization (based on pH and a chemical’s estimated dissociation constant [p*K*_a_] value) and binding of the neutral form.

Absorption occurs at exchange surfaces in contact with the environment (e.g., fish gills or the root cuticle in a plant) or an extension thereof (e.g., the gastrointestinal lumen). For aquatic species, chemical uptake is primarily limited to the unbound fraction in water or the contents of the gastrointestinal tract, whereas in terrestrial plants uptake is limited to chemicals that are mobile in soil water or exist in the gas phase. Chemicals absorbed by the organism are distributed internally by bulk transport (e.g., in blood or in xylem and phloem) and may bind to tissue macromolecules such as lipids and proteins. Chemicals that bind noncovalently remain available for transport out of the organism whereas those that bind covalently or with very high affinity are effectively sequestered.

Passive chemical diffusion across an external exchange surface is inherently bidirectional. Net flux due to diffusion at these exchange surfaces is determined, therefore, by competing rates of uptake and elimination. Additional routes of elimination in fish include excretion of a parent chemical in urine and bile, and biotransformation (Kleinow et al., 2008). Membrane transporters, which have evolved to transport polar molecules across biological membranes, may contribute to the elimination of parent chemicals and their metabolites by specific organs such as the liver and kidney (Ferreira et al., 2014; Luckenbach et al., 2014; Popovic et al., 2014). For plants, the only route of elimination apart from loss across the exchange surfaces is biotransformation. Biotransformation and transport by membrane transporters can saturate at high substrate concentrations and may exhibit competitive inhibition when multiple substrates are present. Many chemicals induce the synthesis of biotransformation enzymes and/or membrane transporters, effectively altering an organism’s capacity for elimination.

Mathematical models that describe the accumulation of neutral organic chemicals in fish and plants have been developed over several decades and are widely used in chemical regulatory programs to support decision making (Arnot & Gobas, 2003, 2004; Paterson et al., 1994; Trapp, 2007; Trapp & Matthies, 1995). Octanol–water partitioning (log *K*_OW_) and proximate composition (i.e., total lipid and water content) are key input parameters required to generate predictions. These models are well supported by empirical data and have been evaluated against data from field sampling efforts and standardized laboratory testing. With simple modifications, models developed for neutral chemicals can be used to predict the bioaccumulation of weak acids and bases if a substantial fraction of total chemical (more than 10%; Armitage et al., 2017) exists internally as the neutral form; in such cases, the contribution of the ionized form to total accumulation is often ignored. Existing data indicate, however, that some highly (more than 99%) ionized chemicals can accumulate in both aquatic and terrestrial biota (Burkhard, 2021; Gredelj et al., 2020; Kierkegaard et al., 2020). In such cases, the simplifying assumption that ionized chemicals do not diffuse across biological membranes is clearly inadequate. In principle, even slow rates of membrane diffusion can support substantial bioaccumulation if exposure is of sufficient duration, elimination is limited, and the chemical has high affinity for tissue constituents. It is imperative, therefore, that predictive models be able to account for these behaviors.

## PROGRESS IN MODELING ACCUMULATION OF IONIZABLE PPCPS IN AQUATIC ORGANISMS

The last decade has seen advances in our ability to predict the accumulation of PPCPs in aquatic invertebrates including the first machine learning approach to predict bioconcentration in *Gammarus pulex* and an approach to characterize pH-dependent uptake by oligochaetes (Karlsson et al., 2017; Miller et al., 2019). It is important to note that whereas the approach by Karlsson et al. (2017) was built on experimental data for PPCPs, the dataset for the machine learning approach by Miller et al. (2019) included a variety of chemicals outside the PPCP domain including pigments, polychlorinated biphenyls, and polyaromatic hydrocarbons, of which approximately 36% were ionized. Meredith-Williams et al. (2012) observed that the pH-corrected liposome–water partition coefficient was a good predictor of bioconcentration factors (BCFs) for aquatic invertebrates (*R*^2^ = 0.83–0.89). A recent study has shown that simple physicochemical parameters related to chemical hydrophobicity (e.g., log *K*_OW_, log *D*) are insufficient to predict accumulation of PPCPs in aquatic invertebrates because they poorly reflect the underlying behavior of ionizable chemicals (Veseli et al., 2022).

Additional advances have been made in predicting the accumulation of ionizable PPCPs in fish. Specifically, the last 10 years have seen significant developments in the parameterization and application of generic, mechanistic mass balance models for describing bioaccumulation of ionizable PPCPs in fish. Measured and empirically derived bioaccumulation parameters for some PPCPs (e.g., steady-state BCFs, gill uptake rate constants, total elimination rate constants, kinetic BCFs; Chen et al., 2017; Zhao et al., 2017) and regression-based estimation approaches (quantitative structure–activity relationships [QSARs]; Fu et al., 2009) also exist but are not considered in our study. Recent advances in mass-balance modeling include the implementation of a simplified version of the gill exchange model for ionizable organic chemicals proposed by Erickson et al. (2006a) within a generic mass balance bioaccumulation model for fish (Armitage et al., 2013). A key feature of the proposed gill exchange model is that the potential influence of differences between bulk water pH and pH within the gills on uptake and elimination kinetics is considered (Armitage et al., 2017). The BCFs predicted by the model described in Armitage et al. (2013) for 129 acids and 62 bases were reasonably well correlated with the empirical data (*R*^2^ = 0.68–0.75) and within a factor of 3 on average. Model evaluations indicated that overall, performance was better for “weakly” ionized chemicals (e.g., acids with p*K*_a_ values of 4 or higher, bases with p*K*_a_ values of 10 or less). However, there was much more uncertainty for “strongly” ionized and permanently charged chemicals.

Biotransformation is a critical determinant of chemical bioaccumulation. Of special interest is the rate of biotransformation, typically expressed as a first-order whole-body rate constant (*k*_B_), relative to competing rates of uptake and other elimination processes. Using a model-based approach, Arnot et al. evaluated in vivo BCFs and measured rates of depuration for approximately 700 organic chemicals to obtain a set of estimated *k*_B_ values (Arnot, Mackay, Parkerton, et al., 2008; Arnot, Mackay, & Bonnell, 2008). The resulting *k*_B_ estimates have been used to develop QSARs that predict *k*_B_ from chemical structure/property information (Arnot et al., 2009; Brown et al., 2012; Kuo & Di Toro, 2013; Mansouri et al., 2018; Papa et al., 2014).

An alternative to the QSAR method for *k*_B_ prediction in fish involves the use of in vitro methods (Krause & Goss, 2020; Nichols et al., 2006, 2009; Trowell et al., 2018). The aim in such studies is to extrapolate a measured rate of in vitro intrinsic clearance to an estimate of *k*_B_ (in vitro–in vivo extrapolation [IVIVE]), taking into account differences in chemical binding in vitro and in tissues (blood and/or liver and/or the fish considered as a whole, depending on the IVIVE approach). With respect to PPCPs, trout live S9 and hepatocyte assays have been used to investigate the biotransformation of pharmaceuticals (Connors et al., 2013; Gomez et al., 2010), fragrance chemicals (Laue et al., 2014), and ionizable surfactants (Chen et al., 2016; Droge et al., 2021). Additional measurements for pharmaceuticals have been obtained using a liver spheroid system (Baron et al., 2017). The utility of in vitro methods for chemicals that undergo slow rates of biotransformation may be limited by the working lifetime of the preparation (Nichols et al., 2021). These methods also fail to account for biotransformation in tissues other than the liver (Saunders et al., 2020).

Lastly, recent observations have stimulated renewed interest in renal clearance as an elimination pathway (Consoer et al., 2014; Ng & Hungerbühler, 2013). Renal clearance is generally ignored in mass balance models for fish because gill elimination is expected to be much greater. However, studies by Consoer et al. (2014) demonstrated the predominance of renal clearance as a mechanism for elimination of perfluorooctanoate (PFOA) in rainbow trout. Modeling studies also suggest that renal clearance plays an important role in elimination of several shorter chain (less than eight perfluorinated carbons) perfluorinated alkyl acids (PFAAs) by fish (Sun et al., 2022). These findings are consistent with earlier research on anionic chemicals such as 2,4-dichlorophenoxyacetic acid (Pritchard & James, 1979) and suggest that active transport to urine mediated by renal organic anion transporters (OATs) plays an important role in the elimination of some anionic substances by fish. Whereas the broader importance of active transport and renal clearance for PPCPs is unclear and relevant empirical data are generally lacking, an approach to model interactions with OATs and renal clearance for PFAAs using in vitro data has been proposed in the literature (Ng & Hungerbühler, 2013) and may be viable for other ionizable PPCPs in the future.

## PROGRESS IN MODELING THE ACCUMULATION OF IONIZABLE PPCPS IN TERRESTRIAL ORGANISMS

In the terrestrial environment, work to date has focused on the accumulation of a select number of ionizable pharmaceuticals by earthworms (Carter et al., 2014, 2016). A first-order one-compartment toxicokinetic model was successfully fitted to the data and allowed the subsequent determination of earthworm BCFs; however, further analysis revealed that previously published QSARs to predict BCFs (see Jager, 1998) performed poorly, which suggested that further model development is required (Carter et al., 2014). In comparison, considerable focus has been on the development, parameterization, and application of models describing bioaccumulation of ionizable PPCPs in plants. The mechanistic description of uptake and redistribution of dissociating electrolytes in plant cells’ organelles by Trapp (2000) and further refined and described by Trapp (2004, 2009), forms the basis of a number of approaches that have been published in the last decade to model the in-plant accumulation of ionizable PPCPs.

Partition coefficients calculated using the cell model are used in several dynamic plant uptake (DPU) models to estimate chemical transport within a plant, accounting for the fact that in typical exposure scenarios, steady-state solutions are not appropriate. For example, the dynamic model for neutral compounds (Trapp, 2015) was modified by González García et al. (2019) to consider additional processes relevant for ionic compounds, such as (1) sorption to proteins, (2) speciation and ion trapping, and (3) phloem transport of weak acids. A similar approach was adopted by Brunetti et al. (2022), who revised their earlier model for neutral compounds (Brunetti et al., 2019), to take into account processes such as chemical dissociation, electrical interaction with plant membranes, and phloem transport to simulate the reactive transport of electrolytes in the soil–plant continuum. The modeling framework connected the cell model to describe processes at a multi-organelle level with a multicompartment DPU model to simulate the reactive transport of electrolytes in the soil and plant (Polesel et al., 2015; Šimůnek et al., 2016; Trapp, 2004). The model outputs confirmed that pH conditions in both the soil and xylem play a crucial role in the uptake and translocation of ionizable chemicals, providing further evidence that we cannot just rely on models developed for neutral compounds.

In an additional level of complexity, integrated models describe chemical fate across different scenarios and compartments (e.g., wastewater/biosolids–soil–crops). For example, Polesel et al. (2015) developed a model to simulate the fate of ionizable PPCPs from human consumption up to the accumulation in the plant following irrigation with wastewater or soil amendment with biosolids. Delli Compagni et al. (2020) have also applied an integrated approach to predict ionizable PPCP fate in different types of wastewater reuse systems. To achieve this, a river water quality model, with extended chemical fate processes accounting for environmental pH on partitioning behavior, was combined with a coupled soil–plant model that described the partitioning of ionizable chemicals between plant tissues and soil, and xylem and phloem flows.

Meanwhile, Prosser et al. (2014) examined the ability of the DPU model and the biosolids-amended Soil Level IV model (BASL4) to predict the concentration of eight PPCPs in plants grown in biosolids-amended soil. In BASL4, ionization of chemicals was accounted for in a number of ways including the replacement of partition coefficients (i.e., organic carbon–water partitioning *K*_OC_, *K*_OW_) and water solubility of the neutral PPCP molecules with distribution ratios (i.e., *D*_OC_, *D*_OW_) and adjusted water solubility to address the speciation that occurs at soil pH. It is also important to acknowledge advances in modeling pesticide exposure, and similarities in the ionogenic nature of pesticides offer promise in utilizing these frameworks for modeling the accumulation of ionizable PPCPs. For example, dynamiCROP, which simulates accumulation into multiple crop types (Fantke et al., 2011), has been shown to model uptake of a wide range of pesticides including ionizable compounds such as azoxystrobin in passion fruit with good accuracy (Juraske et al., 2012).

Pharmaceuticals have been extensively studied in laboratory animals and humans. Mechanistic modeling approaches conceptually similar to those used to simulate ionizable PPCPs in fish have recently been implemented to simulate bioaccumulation in air-breathing organisms (Armitage et al., 2021; Arnot et al., 2022). Preliminary model evaluations indicate similar performance for neutral organics and weak acids and bases, but further comparisons are needed. As with aquatic organisms, greater uncertainty is expected for strongly ionized PPCPs due to the challenges in estimating membrane permeation (e.g., gut uptake efficiencies) and sorption to biological macromolecules (i.e., phospholipids, serum albumin, other proteins).

## FUTURE RESEARCH PRIORITIES

As summarized in the previous sections, the last decade has brought significant advances in our ability to model the accumulation of PPCPs. Further model development is hampered, however, by a lack of mechanistic insights for PPCPs that are predominantly or permanently ionized. To improve predictive bioaccumulation models for such chemicals, targeted experiments across environmentally realistic conditions are needed to address the following questions:

### What are the partitioning and sorption behaviors of strongly ionizing chemicals among species?

Regression equations that predict steady-state BCFs in fish from log *K*_OW_ have been provided by numerous authors (Mackay, 1982; Veith et al., 1979). Implied by this approach is an assumption that octanol is a good surrogate for the biological phases into which organic chemicals partition. Substantial data indicate, however, that octanol is a poor surrogate phase for ionizable organics and tends to underestimate sorption of ions (Escher & Schwarzenbach, 1996). In recent years, sorption data for ionizable chemicals have been obtained for liposomes (membrane phospholipids), serum albumin, structural proteins, and other biologically relevant phases (Allendorf et al., 2019; Bittermann et al., 2016, 2018; Droge, 2018; Droge et al., 2021; Ebert et al., 2020; Goss et al., 2018; Henneberger & Goss, 2021; Henneberger et al., 2016a, 2016b, 2022; Linden et al., 2017). However, empirical data remain scarce compared with the number of ionizable chemicals requiring assessment. Methods to predict key partitioning coefficients and distribution ratios for strongly ionized chemicals have been developed but are computationally demanding and involve proprietary software (e.g., COSMOmic; Bittermann et al., 2016).

The distributional behavior of a drug in mammals is commonly described by its apparent volume of distribution (*V*_D_), which is defined as the sorption capacity of the organism relative to that of blood. The *V*_D_ may be estimated in relatively simple kinetics experiments and, once known, provides an empirical description of internal partitioning regardless of the basis for this behavior. This concept may have special utility for modeling the accumulation of ionized chemicals in fish (Brooks & Steele, 2018; Nichols et al., 2009). In fact, Zhang et al. (2022) recently found that human *V*_D_ values predicted kinetic-based BCFs of pharmaceuticals in zebrafish to a greater extent than *D*_OW_ or *D*_liposome-water_. Presently, there are very few measured *V*_D_ values for nonmammalian species.

Options to replace log *K*_OW_ in plant accumulation modeling have also been proposed. In a recent study, González García et al. (2019) estimated sorption to plant proteins using the human serum albumin–water partition ratio (*K*_HSA_). Conceptual frameworks for addressing the different sorption behaviors exhibited by ionizable chemicals are therefore well established and have been tested. Nevertheless, significant challenges to reliably predict the partitioning of ions remain. It is also important to recognize that such partition coefficient/distribution ratio approaches are assumed to apply across species, but further evaluations for PPCPs and other ionizable organic contaminants are warranted.

### How does membrane permeability of ions influence bioaccumulation of PPCPs?

The membrane permeability of ions is closely related to the previous question of sorption, with the permeability (*P*) of neutral chemicals calculated using the membrane partition coefficient (*K*), the diffusion coefficient (*D*), and the membrane thickness (Δ*x*; Trapp, 2004). Because the membrane partition coefficient, *K*, of neutral compounds is closely related to the log *K*_OW_, regressions of *P* versus the log *K*_OW_ have been proposed (Grayson & Kleier, 1990; Trapp, 2004). New regressions for ions based, for example, on *K*_lipw_ (or *D*_lipw_) are yet to be developed and tested. In any case, due to the strong dipole moment, ions permeate much more slowly (1000–10 000 times; Kleier, 1988) through membranes. Moreover, membranes are electrically charged, and thus membrane permeation of organic ions is correctly described by the Nernst–Planck equation and not by Fick’s first law of diffusion (Trapp, 2004). The need to better understand membrane permeability of ionized chemicals is well illustrated by the observed behavior of PFAS in fish. If ionized chemicals did not diffuse across gill membranes, the uptake of such chemicals from water would be negligible. Instead, even though gill uptake efficiencies are low (less than 1% for perfluorooctanesulfonic acid and PFOA in large rainbow trout; Consoer et al., 2016), they are sufficient to support substantial accumulation (Martin et al., 2003).

### To what extent are salts and associated complexes with PPCPs influencing bioaccumulation?

To date, modeling approaches have rarely considered that living cells are a “salty” environment (ionic strength [*I*] of cytosol 0.3 M). Ionic strength is a descriptor for the overall concentration of charges in a solution and is therefore important in determining the activity coefficient (γ) for neutral molecules and ions. Because partition coefficients for ions increase with ionic strength, changes in chemical activity can lead to chemical enrichment inside the cells and thus alter bioaccumulation predictions (Trapp et al., 2010). For example, an activity coefficient of 0.3 for bivalent ions (when *I* = 0.3 M, charge (*z*) = 2) suggests chemical enrichment of a factor 3.3 compared with dilute water (Trapp et al., 2010). Moreover, all electrolytes can form salts and complexes involving counter ions, as described by the law of mass action. These complexes are electrically neutral and will behave differently from their ionic form. Few examples have been studied (e.g., tributyl tin; Arnold et al., 1997), and to date it remains undetermined as to the extent that complexation affects the properties of ionizable PPCPs, and therefore related processes such as sorption and membrane permeability. This fundamental knowledge is needed to understand and account for salts and complexes in future bioaccumulation modeling efforts.

### How do biotransformation and other elimination processes vary within and among species?

Biotransformation and other elimination processes are of particular importance when considering the bioaccumulation of strongly ionized chemicals given that the uptake of such chemicals is typically slower than that of neutral molecules. In most cases, biotransformation results in chemical products that possess less potential to accumulate than the parent chemical from which they derive; however, some exceptions are known to exist (Chen et al., 2017; Droge et al., 2021). In some cases, a comprehensive assessment of bioaccumulation potential may require knowledge of both biotransformation rates and the behavior of specific metabolic products.

Fish biotransformation QSARs have been largely trained on *k*_B_ estimates for neutral organic chemicals, obtained by modeling measured bioaccumulation data. Currently, uncertainties relating to uptake and tissue binding of strongly ionized and permanently charged chemicals make it difficult to estimate *k*_B_ values from in vivo data. However, an accurate bioaccumulation model for these chemicals would provide for this possibility. As such models become available, they should be used to generate new *k*_B_ estimates from in vivo data as a means of improving existing biotransformation QSARs. Similarly, the development and use of IVIVE methods for *k*_B_ estimation in fish has focused on neutral, hydrophobic chemicals. Substantial uncertainty exists in the use of such methods for strongly ionized chemicals. Of special concern is a need to accurately predict chemical binding in vitro and in fish tissues. Thus, the research needed to apply IVIVE methods to these chemicals overlaps substantially with that required to estimate in vivo *k*_B_ values. Read-across from mammalian studies offers promise to understand the types of pharmaceutical metabolites that are to be expected in fish and other aquatic organisms, but does not provide quantitative organism-specific biotransformation parameters (*k*_B_ or the whole-body elimination half-life) for model development and improvement. There is a special need to characterize rates of biotransformation in widely used experimental fish models (e.g., zebrafish) as well as species of commercial importance and those of concern from a conservation perspective.

Additional research should be focused on biotransformation in understudied organisms, such as aquatic invertebrates, which are known to metabolize a range of PPCPs (Chen et al., 2017; Fu et al., 2018; Jeon et al., 2013; Miller et al., 2017; Rösch et al., 2016). There is a clear need to assess biotransformation dynamics across diverse invertebrate taxa, because even closely related species such as *G. pulex* and *Hyallela azteca* may exhibit different rates of biotransformation (Fu et al., 2018) and resulting biotransformation products (Jeon et al., 2013). Models that can be used to estimate in vivo biotransformation rates in invertebrates have been provided (Ashauer et al., 2012; Ratier et al., 2021). Conceptually, therefore, methods used to develop fish biotransformation QSARs for fish could be applied to invertebrates. Given the substantial data requirements for development of a biotransformation QSAR, however, the more useful approach, at least in the short term, may be the development of in vitro biotransformation assays and associated IVIVE procedures.

The “green liver” concept (Sandermann, 1999) has been proposed to relate the metabolic processes of plants and animals. A difference between animals and plants is that the latter excrete complexed metabolites into vacuoles or cell walls, and hence metabolites are not removed from the plant, which may be the reason for the high number of PPCP metabolites identified in plants (Carter et al., 2018; Macherius et al., 2014; Riemenschneider et al., 2017). Analytically, such complexes and bound residues are a challenge, and are thus rarely identified, which limits the availability of empirical data for model development (Kästner et al., 2014; Sandermann, 2004). Moreover, the available data show high variability (Fantke & Juraske, 2013). Focused efforts are therefore required to develop approaches to model biotransformation of ionizable PPCP in plants and the sequestration of metabolites in cell walls and vacuoles.

### Are bioaccumulation modeling efforts currently focused on chemicals and species with key data gaps and risk profiles?

Extensive efforts have aimed to prioritize PPCPs for future study, as noted elsewhere in this special series on priority research questions. Such exercises are pragmatic given the diversity of chemical classes within PPCPs, and the limited availability of information on their environmental fate, effects, and risks. For example, Nendza et al. (2018) screened data sources for aquatic bioaccumulation data. All acids and bases in this evaluation that fulfilled the REACH B criterion (BCF greater than 2000 L/kg; ECHA European Chemical Agency, 2017) were either mostly nonionized under the tested conditions or PFAS. Bioaccumulation test data for mostly or fully ionized chemicals remain relatively scarce, however, in part due to the expectation of low bioaccumulation potential. Furthermore, a low bioaccumulation potential does not negate potential environmental risk, particularly when biological activities of many PPCPs are more targeted and potent than most industrial chemicals. Ankley et al. (2007) highlighted problems associated with using usage tonnage-based cut-off values to assess and manage PPCPs, and recently published research has demonstrated that low concentrations in aquatic and terrestrial systems can elicit sublethal effects in nontarget organisms (Bertram et al., 2022; Carter et al., 2015; Horký et al., 2021; Martin et al., 2019), with a potential for wider ecosystem effects. It is therefore important that future bioaccumulation models be relevant for chemicals with a broad range of physicochemical properties and not just those expected to accumulate to the greatest extent.

Additional focus needs to be placed on understudied organisms. It is important for future research efforts to consider other nontarget organisms such as aquatic and terrestrial invertebrates (Karlsson et al., 2017; Miller et al., 2015). This is particularly important given the potential for trophic transfer in diverse ecosystems (Du et al., 2014; Haddad et al., 2018) and scenarios, such as nectar and pollen to bees (Carter et al., 2020).

### What are the key sources of uncertainty for PPCP bioaccumulation modeling?

Insufficient process descriptions lead to model uncertainty. For neutral PPCPs, the principal uncertainties relate to biotransformation (rate and behavior of metabolic products) and the behavior of extremely hydrophobic (log *K*_OW_ greater than 9) chemicals. For weak acids and bases (p*K*_a_ 4–10), the principal modeling uncertainties relate to binding of the ionized chemical species, the potential role of membrane transporters in chemical elimination, biotransformation, and the impact of pH gradients that are often undescribed or variable. In plants, for example, there can be a large pH gradient between xylem (pH 4.5–5.5) and phloem (pH 8), and between cytosol (pH 7.4) and vacuoles (pH 4.5–5.5). Ion trapping effects may therefore add considerable uncertainty to the prediction of translocation and accumulation (Briggs et al., 1987; Delli Compagni et al., 2020). Similarly, the elimination of metabolically produced acid at fish gills acidifies the microenvironment at the gill surface (Erickson et al., 2006b); however, the generalizability of this phenomena among different fish species, chemicals, and exposure settings is largely unknown. In the design of both experimental studies and field sampling efforts, it is important to consider how such factors could inform model development and to measure and report appropriate parameters. Often, experiments are not described in the necessary detail to simulate observed effects with mathematical models (Doucette et al., 2018; Trapp, 2015).

Ionizable compounds are, by definition, present as more than one molecular species, and the behavior of the neutral and ionized forms can differ substantially. There are thus more processes to consider, and more equations and parameters required for their description than for neutral compounds. This alone leads to an increase in variability and uncertainty in modeled fate predictions. However, it is important to acknowledge that this variation is not limited to the model world: it is also found in the results of experimental studies in which PPCP accumulation shows wide variance, and a significant challenge remains as to how to account for this (Delli Compagni et al., 2020; Polesel et al., 2015). Additional variability in reported empirical BCF values in plants is introduced by different definitions of the soil concentration when one is deriving BCFs (see Trapp & Eggen, 2013, Supporting Information, Table 6).

For strongly ionized and permanently charged PPCPs, the principal modeling uncertainties relate to diffusion of ionized chemical species across membranes, binding of ionized species to tissue macromolecules, biotransformation, and the potential role of membrane transporters. With respect to these latter elimination processes, existing methods may not provide the sensitivity needed to measure low but “relevant” rates of activity, which are nonetheless important in terms of regulating bioaccumulation. Because uptake rates for ions are relatively slow, short-term laboratory experiments may be insufficient to reliably measure bioaccumulation. It is thus necessary to screen for bioaccumulation of PPCPs in field-collected organisms from both aquatic and terrestrial food webs. As presented, significant progress has been made in the last decade with respect to the development of bioaccumulation models to better account for processes specific to ionizable PPCPs. It is important that research now be tailored toward capitalizing on these developments to address the remaining sources of model uncertainty, which would pave the way for a suite of robust models for a broad spectrum of ionizable PPCPs.

## Data Availability

Data sharing is not applicable to this article because no datasets were generated or analyzed during the present study. Data, associated metadata, and calculation tools are available from the corresponding author (l.j.carter@leeds.ac.uk).

## References

[R1] Allendorf F, Berger U, Goss KU, & Ulrich N (2019). Partition coefficients of four perfluoroalkyl acid alternatives between bovine serum albumin (BSA) and water in comparison to ten classical perfluoroalkyl acids. Environmental Science: Processes & Impacts, 21(11), 1852–1863. 10.1039/C9EM00290A 31475719

[R2] Ankley GT, Brooks BW, Huggett DB, & Sumpter JP (2007). Repeating history: Pharmaceuticals in the environment. Environmental Science and Technology, 41(24), 8211–8217. 10.1021/ES072658J/ASSET/ES072658J.FP.PNG_V03 18200843

[R3] Armitage JM, Arnot JA, Wania F, & Mackay D (2013). Development and evaluation of a mechanistic bioconcentration model for ionogenic organic chemicals in fish. Environmental Toxicology and Chemistry, 32(1), 115–128. 10.1002/etc.2020 23023933

[R4] Armitage JM, Erickson RJ, Luckenbach T, Ng CA, Prosser RS, Arnot JA, Schirmer K, & Nichols JW (2017). Assessing the bioaccumulation potential of ionizable organic compounds: Current knowledge and research priorities. Environmental Toxicology and Chemistry, 36(4), 882–897. 10.1002/etc.3680 27992066 PMC6172661

[R5] Armitage JM, Hughes L, Sangion A, & Arnot JA (2021). Development and intercomparison of single and multicompartment physiologically-based toxicokinetic models: Implications for model selection and tiered modeling frameworks. Environment International, 154, 106557. 10.1016/J.ENVINT.2021.106557 33892222

[R6] Arnold CG, Weidenhaupt A, David MM, Muller SR, Haderlein SB, & Schwarzenbach RP (1997). Aqueous speciation and 1-octanol-water partitioning of tributyl-and triphenyltin: Effect of pH and ion composition. Environmental Science & Technology, 31, 2596–2602.

[R7] Arnot JA, & Gobas FAPC (2003). A Generic QSAR for assessing the bioaccumulation potential of organic chemicals in aquatic food webs. QSAR & Combinatorial Science, 22(3), 337–345. 10.1002/qsar.200390023

[R8] Arnot JA, & Gobas FAPC (2004). Special Issue honoring Don Mackay. A food web bioaccumulation model for organic chemicals in aquatic ecosystems. Environmental Toxicology and Chemistry, 23(10), 2343–2355. http://laws.justice.gc.ca/en/C-15.31 15511097 10.1897/03-438

[R9] Arnot JA, Mackay D, & Bonnell M (2008). Estimating metabolic biotransformation rates in fish from laboratory data. Environmental Toxicology and Chemistry, 27(2), 341–351. 10.1897/07-310R.1 18348640

[R10] Arnot JA, Mackay D, Parkerton TF, & Bonnell M (2008). A database of fish biotransformation rates for organic chemicals. Environmental Toxicology and Chemistry, 27(11), 2263–2270. 10.1897/08-058.1 18522452

[R11] Arnot JA, Meylan W, Tunkel J, Howard PH, Mackay D, Bonnell M, & Boethling RS (2009). A quantitative structure-activity relationship for predicting metabolic biotransformation rates for organic chemicals in fish. Environmental Toxicology and Chemistry, 28(6), 1168–1177. 10.1897/08-289.1 19152232

[R12] Arnot JA, Toose L, Armitage JM, Embry M, Sangion A, & Hughes L (in press). Special Series. A weight of evidence approach for bioaccumulation assessment. Integrated Environmental Assessment and Management. 10.1002/ieam.4583 35049141

[R13] Ashauer R, Hintermeister A, O’Connor I, Elumelu M, Hollender J, & Escher BI (2012). Significance of xenobiotic metabolism for bioaccumulation kinetics of organic chemicals in *Gammarus pulex*. Environmental Science & Technology, 46(6), 3498–3508. 10.1021/es204611h 22321051 PMC3308200

[R14] Baron MG, Mintram KS, Owen SF, Hetheridge MJ, Moody AJ, Purcell WM, Jackson SK, & Jha AN (2017). Pharmaceutical metabolism in fish: Using a 3-D hepatic in vitro model to assess clearance. PLoS One, 12(1), e0168837. 10.1371/JOURNAL.PONE.0168837 28045944 PMC5207725

[R15] Bertram MG, Martin JM, McCallum ES, Alton LA, Brand JA, Brooks BW, Cerveny D, Fick J, Ford AT, Hellström G, Michel-angeli M, Nakagawa S, Polverino G, Saaristo M, Sih A, Tan H, Tyler CR, Wong BBM, & Brodin T (2022). Frontiers in quantifying wildlife behavioural responses to chemical pollution. Biological Reviews of the Cambridge Philosophical Society, 97(37), 1346–1364. 10.1111/BRV.12844 35233915 PMC9543409

[R16] Bittermann K, Linden L, & Goss KU (2018). Screening tools for the bioconcentration potential of monovalent organic ions in fish. Environmental Science: Processes and Impacts, 20(5), 845–853. 10.1039/C8EM00084K 29714798

[R17] Bittermann K, Spycher S, & Goss KU (2016). Comparison of different models predicting the phospholipid-membrane water partition coefficients of charged compounds. Chemosphere, 144, 382–391. 10.1016/J.CHEMOSPHERE.2015.08.065 26383265

[R18] Boxall ABA, Rudd MA, Brooks BW, Caldwell DJ, Choi K, Hickmann S, Innes E, Ostapyk K, Staveley JP, Verslycke T, Ankley GT, Beazley KF, Belanger SE, Berninger JP, Carriquiriborde P, Coors A, DeLeo PC, Dyer SD, Ericson JF, & Van Der Kraak G (2012). Pharmaceuticals and personal care products in the environment: What are the big questions? Environmental Health Perspectives, 120(9), 1221–1229. 10.1289/ehp.1104477 22647657 PMC3440110

[R19] Briggs GG, Rigitano RLO, & Bromilow RH (1987). Physico-chemical factors affecting uptake by roots and translocation to shoots of weak acids in barley. Pesticide Science, 19(2), 101–112. 10.1002/PS.2780190203

[R20] Brinch A, Jensen AA, & Christensen F (2018). Risk assessment of fluorinated substances in cosmetic products. The Danish Environmental Protection Agency.

[R21] Brooks BW, Chambliss CK, Stanley JK, Ramirez A, Banks KE, Johnson RD, & Lewis RJ (2005). Determination of select anti-depressants in fish from an effluent-dominated stream. Environmental Toxicology and Chemistry, 24(2), 464–469. 10.1897/04-081R.1 15720009

[R22] Brooks BW, & Steele WB (2018). Ecotoxicological perspectives on healthcare and the environment. In Boxall ABA & Kookana RS (Eds.), Healthcare and environmental contaminants (pp. 41–66). Elsevier.

[R23] Brown TN, Arnot JA, & Wania F (2012). Iterative fragment selection: A group contribution approach to predicting fish biotransformation half-lives. Environmental Science & Technology, 46(15), 8253–8260. 10.1021/ES301182A/SUPPL_FILE/ES301182A_SI_001.PDF 22779755

[R24] Brunetti G, Kodešová R, & Šimůnek J (2019). Modeling the translocation and transformation of chemicals in the soil-plant continuum: A dynamic plant uptake module for the HYDRUS model. Water Resources Research, 55(11), 8967–8989. 10.1029/2019WR025432

[R25] Brunetti G, Kodešová R, Švecová H, Fér M, Nikodem A, Klement A, Grabic R, & Šimůnek J (2022). A novel multiscale biophysical model to predict the fate of ionizable compounds in the soil-plant continuum. Journal of Hazardous Materials, 423, 127008. 10.1016/J.JHAZMAT.2021.127008 34844334

[R26] Burkhard LP (2021). Evaluation of published bioconcentration factor (BCF) and bioaccumulation factor (BAF) data for per- and polyfluoroalkyl substances across aquatic species. Environmental Toxicology and Chemistry, 40(6), 1530–1543. 10.1002/ETC.5010 33605484

[R27] Carter LJ, Agatz A, Kumar A, & Williams M (2020). Translocation of pharmaceuticals from wastewater into beehives. Environment International, 134, 105248. 10.1016/J.ENVINT.2019.105248 31711020

[R28] Carter LJ, Garman CD, Ryan J, Dowle A, Bergstrom E, Thomas-Oates J, & Boxall ABA (2014). Fate and uptake of pharmaceuticals in soil-earthworm systems. Environmental Science & Technology, 48(10), 5955–5963. 10.1021/es500567w 24762061 PMC4041664

[R29] Carter LJ, Ryan JJ, & Boxall ABA (2016). Effects of soil properties on the uptake of pharmaceuticals into earthworms. Environmental Pollution, 213, 922–931. 10.1016/j.envpol.2016.03.044 27049789 PMC4894142

[R30] Carter LJ, Williams M, Boettcher C, & Kookana RS (2015). Uptake of pharmaceuticals influences plant development and affects nutrient and hormone homeostases. Environmental Science & Technology, 49(20), 12509–12518. 10.1021/acs.est.5b03468 26418514

[R31] Carter LJ, Williams M, Martin S, Kamaludeen SPB, & Kookana RS (2018). Sorption, plant uptake and metabolism of benzodiazepines. Science of the Total Environment, 628–629, 18–25. 10.1016/j.scitotenv.2018.01.337 29428856

[R32] Chen F, Gong Z, & Kelly BC (2017). Bioaccumulation behavior of pharmaceuticals and personal care products in adult zebrafish (Danio rerio): Influence of physical-chemical properties and biotransformation. Environmental Science and Technology, 51(19), 11085–11095. 10.1021/ACS.EST.7B02918/SUPPL_FILE/ES7B02918_SI_001.PDF 28853873

[R33] Chen Y, Hermens JLM, Jonker MTO, Arnot JA, Armitage JM, Brown T, Nichols JW, Fay KA, & Droge STJ (2016). Which molecular features affect the intrinsic hepatic clearance rate of ionizable organic chemicals in fish. Environmental Science and Technology, 50(23), 12722–12731. 10.1021/ACS.EST.6B03504 27934284

[R34] Connors KA, Du B, Fitzsimmons PN, Hoffman AD, Chambliss CK, Nichols JW, & Brooks BW (2013). Comparative pharmaceutical metabolism by rainbow trout (Oncorhynchus mykiss) liver S9 fractions. Environmental Toxicology and Chemistry, 32(8), 1810–1818. 10.1002/ETC.2240 23606059

[R35] Consoer DM, Hoffman AD, Fitzsimmons PN, Kosian PA, & Nichols JW (2014). Toxicokinetics of perfluorooctanoate (PFOA) in rainbow trout (Oncorhynchus mykiss). Aquatic Toxicology, 156, 65–73. 10.1016/J.AQUATOX.2014.07.022 25150511

[R36] Consoer DM, Hoffman AD, Fitzsimmons NN, Kosian PA, & Nichols JW (2016). Toxicokinetics of perfluorooctane sulfonate in rainbow trout (Oncorhynchus mykiss). Environmental Toxicology and Chemistry, 35(3), 717–727. 10.1002/ETC.3230 26332333

[R37] Daughton CG, & Brooks BW (2011). Active pharmaceutical ingredients and aquatic organisms. In Beyer N & Meador J (Eds.), Environmental contaminants in biota: Interpreting tissue concentrations (2nd ed., pp. 287–347). Taylor & Francis. https://www.semanticscholar.org/paper/%22Active-Pharmaceutical-Ingredients-and-Aquatic-in%3A-Daughton/c1743649b4711d113cc52d08068506694b6c3fb7

[R38] Delli Compagni R, Gabrielli M, Polesel F, Turolla A, Trapp S, Vezzaro L, & Antonelli M (2020). Risk assessment of contaminants of emerging concern in the context of wastewater reuse for irrigation: An integrated modelling approach. Chemosphere, 242, 125185. 10.1016/J.CHEMOSPHERE.2019.125185 31689637

[R39] Doucette WJ, Shunthirasingham C, Dettenmaier EM, Zaleski RT, Fantke P, & Arnot JA (2018). A review of measured bioaccumulation data on terrestrial plants for organic chemicals: Metrics, variability, and the need for standardized measurement protocols. Environmental Toxicology and Chemistry, 37(1), 21–33. 10.1002/etc.3992 28976607

[R40] Droge STJ (2018). Membrane–water partition coefficients to aid risk assessment of perfluoroalkyl anions and alkyl sulfates. Environmental Science and Technology, 53(2), 760–770. 10.1021/acs.est.8b05052 30572703

[R41] Droge STJ, Armitage JM, Arnot JA, Fitzsimmons PN, & Nichols JW (2021). Environmental toxicology biotransformation potential of cationic surfactants in fish assessed with rainbow trout liver S9 Fractions. Environmental Toxicology and Chemistry, 40(11), 3123–3136. 10.1002/etc.5189 34379820 PMC9187044

[R42] Du B, Haddad SP, Luek A, Scott WC, Saari GN, Kristofco LA, Connors KA, Rash C, Rasmussen JB, Chambliss CK, & Brooks BW (2014). Bioaccumulation and trophic dilution of human pharmaceuticals across trophic positions of an effluent-dependent wadeable stream. Philosophical Transactions of the Royal Society, B: Biological Sciences, 369(1656), 20140058. 10.1098/RSTB.2014.0058 PMC4213599 25313153

[R43] Ebert A, Allendorf F, Berger U, Goss KU, & Ulrich N (2020). Membrane/water partitioning and permeabilities of perfluoroalkyl acids and four of their alternatives and the effects on toxicokinetic behavior. Environmental Science & Technology, 54(8), 5051–5061. 10.1021/ACS.EST.0C00175/SUPPL_FILE/ES0C00175_SI_001.PDF 32212724

[R44] Erickson RJ, McKim JM, Lien GJ, Hoffman AD, & Batterman SL (2006a). Uptake and elimination of ionizable organic chemicals at fish gills: I. Model formulation, parameterization, and behavior. Environmental Toxicology and Chemistry, 25(6), 1512–1521. 10.1897/05-358R.1 16764469

[R45] Erickson RJ, McKim JM, Lien GJ, Hoffman AD, & Batterman SL (2006b). Uptake and elimination of ionizable organic chemicals at fish gills: II. Observed and predicted effects of ph, alkalinity, and chemical properties. Environmental Toxicology and Chemistry, 25(6), 1522–1532. 10.1897/05-359R.1 16764470

[R46] Escher BI, & Schwarzenbach RP (1996). Partitioning of substituted phenols in liposome–water, biomembrane–water, and octanol–water systems. Environmental Science and Technology, 30(1), 260–270. 10.1021/ES9503084

[R47] Fantke P, & Juraske R (2013). Variability of pesticide dissipation half-lives in plants. Environmental Science and Technology, 47(8), 3548–3562. 10.1021/ES303525X/SUPPL_FILE/ES303525X_SI_001.XLSX 23521068

[R48] Fantke P, Juraske R, Antón A, Friedrich R, & Jolliet O (2011). Dynamic multicrop model to characterize impacts of pesticides in food. Environmental Science & Technology, 45(20), 8842–8849. 10.1021/ES201989D/SUPPL_FILE/ES201989D_SI_001.PDF 21905656

[R49] Ferreira M, Costa J, & Reis-Henriques MA (2014). ABC transporters in fish species: A review. Frontiers in Physiology, 5, 266. 10.3389/FPHYS.2014.00266/BIBTEX 25101003 PMC4106011

[R50] Franco A, Ferranti A, Davidsen C, & Trapp S (2010). An unexpected challenge: Ionizable compounds in the REACH chemical space. International Journal of Life Cycle Assessment, 15, 321–325. 10.1007/s11367-010-0165-6

[R51] Fu Q, Rösch A, Fedrizzi D, Vignet C, & Hollender J (2018). Bioaccumulation, Biotransformation, and synergistic effects of binary fungicide mixtures in Hyalella azteca and Gammarus pulex: How different/similar are the two species? Environmental Science and Technology, 52(22), 13491–13500. 10.1021/ACS.EST.8B04057/SUPPL_FILE/ES8B04057_SI_001.PDF 30298730

[R52] Fu W, Franco A, & Trapp S (2009). Methods for estimating the bioconcentration factor of ionizable organic chemicals. Environmental Toxicology and Chemistry, 28(7), 1372–1379. 10.1897/08-233.1 19245273

[R53] Fujii Y, Harada KH, & Koizumi A (2013). Occurrence of perfluorinated carboxylic acids (PFCAs) in personal care products and compounding agents. Chemosphere, 93(3), 538–544. 10.1016/J.CHEMOSPHERE.2013.06.049 23932147

[R54] Gomez CF, Constantine L, & Huggett DB (2010). The influence of gill and liver metabolism on the predicted bioconcentration of three pharmaceuticals in fish. Chemosphere, 81(10), 1189–1195. 10.1016/J.CHEMOSPHERE.2010.09.043 20980039

[R55] González García M, Fernández-López C, Polesel F, & Trapp S (2019). Predicting the uptake of emerging organic contaminants in vegetables irrigated with treated wastewater—Implications for food safety assessment. Environmental Research, 172, 175–181. 10.1016/J.ENVRES.2019.02.011 30782537

[R56] Goss KU, Bittermann K, Henneberger L, & Linden L (2018). Equilibrium biopartitioning of organic anions–A case study for humans and fish. Chemosphere, 199, 174–181. 10.1016/J.CHEMOSPHERE.2018.02.026 29438944

[R57] Grayson BT, & Kleier DA (1990). Phloem mobility of xenobiotics. IV. Modelling of pesticide movement in plants. Pesticide Science, 30(1), 67–79. 10.1002/PS.2780300108

[R58] Gredelj A, Polesel F, & Trapp S (2020). Model-based analysis of the uptake of perfluoroalkyl acids (PFAAs) from soil into plants. Chemosphere, 244:125534. 10.1016/J.CHEMOSPHERE.2019.125534 32050335

[R59] Haddad SP, Luek A, Scott WC, Saari GN, Burket SR, Kristofco LA, Corrales J, Rasmussen JB, Chambliss CK, Luers M, Rogers C, & Brooks BW (2018). Spatio-temporal bioaccumulation and trophic transfer of ionizable pharmaceuticals in a semi-arid urban river influenced by snowmelt. Journal of Hazardous Materials, 359, 231–240. 10.1016/J.JHAZMAT.2018.07.063 30036753

[R60] Henneberger L, & Goss KU (2021). Environmental sorption behavior of ionic and ionizable organic chemicals. Reviews of Environmental Contamination and Toxicology, 253, 43–64. 10.1007/398_2019_37 31748892

[R61] Henneberger L, Goss KU, & Endo S (2016a). Equilibrium sorption of structurally diverse organic ions to bovine serum albumin. Environmental Science & Technology, 50(10), 5119–5126. 10.1021/ACS.EST.5B06176/SUPPL_FILE/ES5B06176_SI_001.PDF 27098963

[R62] Henneberger L, Goss KU, & Endo S (2016b). Partitioning of organic ions to muscle protein: Experimental data, modeling, and implications for in vivo distribution of organic ions. Environmental Science and Technology, 50(13), 7029–7036. 10.1021/ACS.EST.6B01417/SUPPL_FILE/ES6B01417_SI_001.PDF 27265315

[R63] Henneberger L, Klüver N, Mühlenbrink M, & Escher B (2022). Trout and human plasma protein binding of selected pharmaceuticals informs the fish plasma model. Environmental Toxicology and Chemistry, 41(3), 559–568. 10.1002/ETC.4934 33201515

[R64] Horký P, Grabic R, Grabicová K, Brooks BW, Douda K, Slavik O, Hubená P, Santos EMS, & Randák T (2021). Methamphetamine pollution elicits addiction in wild fish. Journal of Experimental Biology, 224(13), jeb242145. 10.1242/JEB.242145/270755 34229347

[R65] Jager T (1998). Mechanistic approach for estimating bioconcentration of organic chemicals in earthworms (Oligochaeta). Environmental Toxicology and Chemistry, 17(10), 2080–2090. 10.1002/ETC.5620171026

[R66] Jeon J, Kurth D, & Hollender J (2013). Biotransformation pathways of biocides and pharmaceuticals in freshwater crustaceans based on structure elucidation of metabolites using high resolution mass spectrometry. Chemical Research in Toxicology, 26(3), 313–324. 10.1021/TX300457F 23391280

[R67] Juraske R, Fantke P, Ramírez ACR, & González A (2012). Pesticide residue dynamics in passion fruits: Comparing field trial and modelling results. Chemosphere, 89(7), 850–855. 10.1016/J.CHEMOSPHERE.2012.05.007 22673401

[R68] Karlsson MV, Carter LJ, Agatz A, & Boxall ABA (2017). Novel approach for characterizing pH-dependent uptake of ionizable chemicals in aquatic organisms. Environmental Science & Technology, 51(12), 6965–6971. 10.1021/acs.est.7b01265 28553715

[R69] Kästner M, Nowak KM, Miltner A, Trapp S, & Schäffer A (2014). Classification and modelling of nonextractable residue (NER) formation of xenobiotics in soil–A synthesis. Critical Reviews in Environmental Science and Technology, 44(19), 2107–2171. 10.1080/10643389.2013.828270

[R70] Kierkegaard A, Chen C, Armitage JM, Arnot JA, Droge S, & McLachlan MS (2020). Tissue distribution of several series of cationic surfactants in rainbow trout (Oncorhynchus mykiss) following exposure via water. Environmental Science & Technology, 54(7), 4190–4199. 10.1021/ACS.EST.9B07600/ASSET/IMAGES/LARGE/ES9B07600_0003.JPEG 32062967 PMC7343282

[R71] Kleier DA (1988). Phloem mobility of xenobiotics I. Mathematical model unifying the weak acid and intermediate permeability theories. Plant Physiology, 86, 803–810. 16665992 10.1104/pp.86.3.803 PMC1054574

[R72] Kleinow K, Nichols J, Hayton W, McKim J, & Barron M (2008). Toxicokinetics in fishes. In Di Giulio RT & Hinton DE (Eds.), The toxicology of fishes (pp. 55–152). Taylor & Francis. 10.1201/9780203647295.CH3

[R73] Krause S, & Goss KU (2020). Comparison of a simple and a complex model for BCF prediction using in vitro biotransformation data. Chemosphere, 256, 127048. 10.1016/J.CHEMOSPHERE.2020.127048 32446001

[R74] Kuo DTF, & Di Toro DM (2013). A reductionist mechanistic model for bioconcentration of neutral and weakly polar organic compounds in fish. Environmental Toxicology and Chemistry, 32(9), 2089–2099. 10.1002/ETC.2283 23703865

[R75] Laue H, Gfeller H, Jenner KJ, Nichols JW, Kern S, & Natsch A (2014). Predicting the bioconcentration of fragrance ingredients by rainbow trout using measured rates of in vitro intrinsic clearance. Environmental Science & Technology, 48(16), 9486–9495. 10.1021/ES500904H 25058173

[R76] Linden L, Goss KU, & Endo S (2017). 3D-QSAR predictions for bovine serum albumin-water partition coefficients of organic anions using quantum mechanically based descriptors. Environmental Science. Processes & Impacts, 19(3), 261–269. 10.1039/C6EM00555A 28009898

[R77] Luckenbach T, Fischer S, & Sturm A (2014). Current advances on ABC drug transporters in fish. Comparative Biochemistry and Physiology. Toxicology & Pharmacology: CBP, 165, 28–52. 10.1016/J.CBPC.2014.05.002 24858718

[R78] Macherius A, Seiwert B, Schröder P, Huber C, Lorenz W, & Reemtsma T (2014). Identification of plant metabolites of environmental contaminants by UPLC-QToF-MS: The in vitro metabolism of triclosan in horseradish. Journal of Agricultural and Food Chemistry, 62(5), 1001–1009. 10.1021/JF404784Q/ASSET/IMAGES/MEDIUM/JF-2013-04784Q_0007.GIF 24456336

[R79] Mackay D (1982). Correlation of bioconcentration factors. Environmental Science & Technology, 16(5), 274–278. 10.1021/ES00099A008/ASSET/ES00099A008.FP.PNG_V03 22257252

[R80] Manallack DT (2007). The pKa distribution of drugs: Application to drug discovery. Perspectives in Medicinal Chemistry, 1, 25. 10.1177/1177391x0700100003 19812734 PMC2754920

[R81] Mansouri K, Grulke CM, Judson RS, & Williams AJ (2018). OPERA models for predicting physicochemical properties and environmental fate endpoints. Journal of Cheminformatics, 10(1), 1–19. 10.1186/S13321-018-0263-1/FIGURES/1 29520515 PMC5843579

[R82] Martin JM, Bertram MG, Saaristo M, Fursdon JB, Hannington SL, Brooks BW, Burket SR, Mole RA, Deal NDS, & Wong BBM (2019). Antidepressants in surface waters: Fluoxetine influences mosquitofish anxiety-related behavior at environmentally relevant levels. Environmental Science & Technology, 53(10), 6035–6043. 10.1021/ACS.EST.9B00944/SUPPL_FILE/ES9B00944_SI_001.PDF 31034220

[R83] Martin JW, Mabury SA, Solomon KR, & Muir DCG (2003). Bioconcentration and tissue distribution of perfluorinated acids in rainbow trout (Oncorhynchus mykiss). Environmental Toxicology and Chemistry, 22(1), 196–204. 10.1002/ETC.5620220126 12503765

[R84] Meredith-Williams M, Carter LJ, Fussell R, Raffaelli D, Ashauer R, & Boxall ABA (2012). Uptake and depuration of pharmaceuticals in aquatic invertebrates. Environmental Pollution, 165, 250–258. 10.1016/j.envpol.2011.11.029 22226124

[R85] Miller TH, Bury NR, Owen SF, & Barron LP (2017). Uptake, biotransformation and elimination of selected pharmaceuticals in a freshwater invertebrate measured using liquid chromatography tandem mass spectrometry. Chemosphere, 183, 389–400. 10.1016/J.CHEMOSPHERE.2017.05.083 28554023 PMC5476196

[R86] Miller TH, Gallidabino MD, MacRae JI, Owen SF, Bury NR, & Barron LP (2019). Prediction of bioconcentration factors in fish and invertebrates using machine learning. Science of the Total Environment, 648, 80–89. 10.1016/J.SCITOTENV.2018.08.122 30114591 PMC6234108

[R87] Miller TH, McEneff GL, Brown RJ, Owen SF, Bury NR, & Barron LP (2015). Pharmaceuticals in the freshwater invertebrate, *Gammarus pulex*, determined using pulverised liquid extraction, solid phase extraction and liquid chromatography-tandem mass spectrometry. Science of the Total Environment, 511, 153–160. 10.1016/j.scitotenv.2014.12.034 25544334

[R88] Nendza M, Kühne R, Lombardo A, Strempel S, & Schüürmann G (2018). PBT assessment under REACH: Screening for low aquatic bioaccumulation with QSAR classifications based on physic al properties to replace BCF in vivo testing on fish. Science of the Total Environment, 616–617, 97–106. 10.1016/J.SCITOTENV.2017.10.317 29107783

[R89] Newton DW, & Kluza RB (2016). pKa Values of medicinal compounds in pharmacy practice: Annals of Pharmacotherapy, 12(9), 546–554. 10.1177/106002807801200906

[R90] Ng CA, & Hungerbühler K (2013). Bioconcentration of perfluorinated alkyl acids: How important is specific binding? Environmental Science & Technology, 47(13), 7214–7223. 10.1021/ES400981A/SUPPL_FILE/ES400981A_SI_001.PDF 23734664

[R91] Nichols JW, Bonnell M, Dimitrov SD, Escher BI, Han X, & Kramer NI (2009). Bioaccumulation assessment using predictive approaches. Integrated Environmental Assessment and Management, 5(4), 577–597. 10.1897/IEAM-2008-088.1 19775192

[R92] Nichols JW, Hoffman AD, Swintek JA, Droge STJ, & Fitzsimmons PN (2021). Addition of phenylmethylsulfonyl fluoride increases the working lifetime of the trout liver S9 substrate depletion assay, resulting in improved detection of low intrinsic clearance rates. Environmental Toxicology and Chemistry, 40(1), 148–161. 10.1002/ETC.4901 33045099 PMC7901806

[R93] Nichols JW, Schultz IR, & Fitzsimmons PN (2006). In vitro–in vivo extrapolation of quantitative hepatic biotransformation data for fish: I. A review of methods, and strategies for incorporating intrinsic clearance estimates into chemical kinetic models. Aquatic Toxicology, 78(1), 74–90. 10.1016/J.AQUATOX.2006.01.017 16513189

[R94] Papa E, Van Der Wal L, Arnot JA, & Gramatica P (2014). Metabolic biotransformation half-lives in fish: QSAR modeling and consensus analysis. Science of the Total Environment, 470–471, 1040–1046. 10.1016/j.scitotenv.2013.10.068 24239825

[R95] Paterson S, Mackay D, & McFarlane C (1994). A model of organic chemical uptake by plants from soil and the atmosphere. Environmental Science & Technology, 28(13), 2259–2266. 10.1021/ES00062A009 22176043

[R96] Polesel F, Plosz BG, & Trapp S (2015). From consumption to harvest: Environmental fate prediction of excreted ionizable trace organic chemicals. Water Research, 85, 85–98. 10.1016/j.watres.2015.06.033 26210033

[R97] Popovic M, Zaja R, Fent K, & Smital T (2014). Interaction of environmental contaminants with zebrafish organic anion transporting polypeptide, Oatp1d1 (Slco1d1). Toxicology and Applied Pharmacology, 280(1), 149–158. 10.1016/J.TAAP.2014.07.015 25088042

[R98] Pritchard JB, & James MO (1979). Determinants of the renal handling of 2, 4-dichlorophenoxyacetic acid by winter flounder. Journal of Pharmacology and Experimental Therapeutics, 208, 2. 762661

[R99] Prosser RS, Trapp S, & Sibley PK (2014). Modeling uptake of selected pharmaceuticals and personal care products into food crops from biosolids-amended soil. Environmental Science & Technology, 48(19), 11397–11404. 10.1021/es503067v 25207852

[R100] Ramirez AJ, Mottaleb MA, Brooks BW, & Chambliss CK (2007). Analysis of pharmaceuticals in fish using liquid chromatography-tandem mass spectrometry. Analytical Chemistry, 79(8), 3155–3163. 10.1021/AC062215I 17348635

[R101] Ratier A, Lopes C, Geffard O, Babut M 2021. The added value of Bayesian inference for estimating biotransformation rates of organic contaminants in aquatic invertebrates. Aquatic Toxicology, 234, 105811. 10.1016/J.AQUATOX.2021.105811 33812312

[R102] Riemenschneider C, Seiwert B, Goldstein M, Al-Raggad M, Salameh E, Chefetz B, & Reemtsma T (2017). An LC-MS/MS method for the determination of 28 polar environmental contaminants and metabolites in vegetables irrigated with treated municipal wastewater. Analytical Methods, 9(8), 1273–1281. 10.1039/C6AY02984A

[R103] Rösch A, Anliker S, & Hollender J (2016). How biotransformation influences toxicokinetics of azole fungicides in the aquatic invertebrate *Gammarus pulex*. Environmental Science & Technology, 50(13), 7175–7188. 10.1021/ACS.EST.6B01301/SUPPL_FILE/ES6B01301_SI_001.PDF 27232586

[R104] Sandermann H (1999). Plant metabolism of organic xenobiotics. Status and prospects of the ‘green liver’ concept. In Altman A, Ziv M, & Izhar S (Eds.), Current plant science and biotechnology in agriculture (pp. 321–328). Springer. 10.1007/978-94-011-4661-6_74

[R105] Sandermann H (2004). Bound and unextractable pesticidal plant residues: Chemical characterization and consumer exposure. Pest Management Science, 60(7), 613–623. 10.1002/PS.888 15260290

[R106] Saunders LJ, Fitzsimmons PN, Nichols JW, & Gobas FAPC (2020). In vitro-in vivo extrapolation of hepatic and gastrointestinal biotransformation rates of hydrophobic chemicals in rainbow trout. Aquatic Toxicology, 228, 105629. 10.1016/J.AQUATOX.2020.105629 33002683 PMC7962060

[R107] Šimůnek J, Van Genuchten MT, Šejna M, Šimůnek J, & Zone V (2016). Recent developments and applications of the HYDRUS computer software packages. Vadose Zone Journal, 15(7), 1–25. 10.2136/VZJ2016.04.0033

[R108] Sun JM, Kelly BC, Gobas FAPC, & Sunderland EM (2022). A food web bioaccumulation model for the accumulation of per- and polyfluoroalkyl substances (PFAS) in fish: How important is renal elimination? Environmental Science: Processes & Impacts. 10.1039/D2EM00047D PMC9384792 35678632

[R109] Trapp S (2000). Modelling uptake into roots and subsequent translocation of neutral and ionisable organic compounds. Pest Management Science, 56(9), 767–778. 10.1002/1526-4998(200009)56:9&lt;767::AID-PS198&gt;3.3.CO;2-H

[R110] Trapp S (2004). Plant uptake and transport models for neutral and ionic chemicals. Environmental Science and Pollution Research, 11(1), 33–39. 10.1065/espr2003.08.169 15005138

[R111] Trapp S (2007). Fruit Tree model for uptake of organic compounds from soil and air. SAR and QSAR in Environmental Research, 18(4), 367–387. 10.1080/10629360701303693 17514576

[R112] Trapp S (2009). Bioaccumulation of polar and ionizable compounds in plants. In Devillers J (Ed.), Ecotoxicology modeling, emerging topics in ecotoxicology: Principles, approaches and perspectives (pp. 299–353). Springer. https://orbit.dtu.dk/en/publications/bioaccumulation-of-polar-and-ionizable-compounds-in-plants

[R113] Trapp S (2015). Calibration of a plant uptake model with plant- and site-specific data for uptake of chlorinated organic compounds into radish. Environmental Science & Technology, 49(1), 395–402. 10.1021/ES503437P/SUPPL_FILE/ES503437P_SI_001.PDF 25426767

[R114] Trapp S, Franco A, & MacKay D (2010). Activity-based concept for transport and partitioning of ionizing organics. Environmental Science & Technology, 44(16), 6123–6129. 10.1021/ES100509X/SUPPL_FILE/ES100509X_SI_001.PDF 20704208

[R115] Trapp S, & Matthies M (1995). Generic one-compartment model for uptake of organic chemicals by foliar vegetation. Environmental Science & Technology, 29(9), 2333–2338. 10.1021/es00009a027 22280275

[R116] Trapp S, & Eggen T (2013). Simulation of the plant uptake of organophosphates and other emerging pollutants for greenhouse experiments and field conditions. Environmental Science and Pollution Research, 20, 4018–4029. 10.1007/s11356-012-1337-7 23212267

[R117] Trowell JJ, Gobas FAPC, Moore MM, & Kennedy CJ (2018). Estimating the bioconcentration factors of hydrophobic organic compounds from biotransformation rates using rainbow trout hepatocytes. Archives of Environmental Contamination and Toxicology, 75, 295–305. 10.1007/s00244-018-0508-z 29550936

[R118] Veith GD, DeFoe DL, & Bergstedt BV (1979). Measuring and estimating the bioconcentration factor of chemicals in fish. Journal of the Fisheries Board of Canada, 36(9), 1040–1048. 10.1139/F79-146

[R119] Veseli M, Rožman M, Vilenica M, Petrović M, & Previšić A (2022). Bioaccumulation and bioamplification of pharmaceuticals and endocrine disruptors in aquatic insects. Science of the Total Environment, 838, 156208. 10.1016/J.SCITOTENV.2022.156208 35618119

[R120] Wang Z, Walker GW, Muir DCG, & Nagatani-Yoshida K (2020). Toward a global understanding of chemical pollution: A first comprehensive analysis of national and regional chemical inventories. Environmental Science & Technology, 54, 2575–2584. 10.1021/acs.est.9b06379 31968937

[R121] Whitehead HD, Venier M, Wu Y, Eastman E, Urbanik S, Diamond ML, Shalin A, Schwartz-Narbonne H, Bruton TA, Blum A, Wang Z, Green M, Tighe M, Wilkinson JT, Mcguinness S, & Peaslee GF (2021). Fluorinated compounds in North American cosmetics. Environmental Science & Technology Letters, 8, 538–544. 10.1021/acs.estlett.1c00240

[R122] Zhang L, Brooks BW, Liu F, Zhou Z, Li H, & You J (in press). Human apparent volume of distribution predicts bioaccumulation of ionizable organic chemicals in zebrafish embryos. Environmental Science & Technology. 10.1021/ACS.EST.2C03421 35896009

[R123] Zhao JL, Furlong ET, Schoenfuss HL, Kolpin DW, Bird KL, Feifarek DJ, Schwab EA, & Ying GG (2017). Uptake and disposition of select pharmaceuticals by bluegill exposed at constant concentrations in a flow-through aquatic exposure system. Environmental Science & Technology, 51(8), 4434–4444. 10.1021/ACS.EST.7B00604/SUPPL_FILE/ES7B00604_SI_001.PDF 28319370

[R124] ECHA European Chemical Agency. (2017). Guidance on Information Requirements and Chemical Safety Assessment, Chapter R.11: Endpoint specific guidance (PBT/vPvB assessment), version 3.0, June 2017.

